# Not everything in the gallbladder is gallstones: an unusual case of biliary ascariasis

**DOI:** 10.1259/bjrcr.20180123

**Published:** 2019-11-15

**Authors:** Chhaya Keshvala, Leena Naidu

**Affiliations:** 1 Kettering General Hospital NHS Foundation Trust, UK; 2 AOUI Veron, Verona, Italy

## Abstract

A 25-year-old female presented with a sore throat, fever and epigastric pain after coming from India. She had an obstructive pattern of liver function tests and an ultrasound scan abdomen showed features suggestive of acute cholecystitis and pancreatitis, with no obvious gallstones. She made no improvement with intravenous antibiotics. Subsequently, a contrast CT demonstrated a curvilinear structure within a thickened gallbladder and common bile duct. After input from multiple specialities, she had a MR cholangiopancreatography (MRCP) which further confirmed the curvilinear filling defect impacted in the gallbladder neck and proximal common bile duct. Biliary ascariasis with mild pancreatitis was diagnosed and was successfully treated with mebendazole. At one-month outpatient follow-up, her liver function tests and ultrasound scan had returned to normal. This case report discusses the radiological findings seen with biliary ascariasis using a range of imaging modalities. It also highlights the importance of the multidisciplinary team in managing a patient who presents a diagnostic challenge, in order to achieve the best patient outcome.

## Presentation

A 25-year-old female of Indian origin presented with a 2-week history of sore throat and fever. She had no significant past medical or family history and was not on any regular medications. Having completed a course of amoxicillin in the community with no improvement, she was started on co-amoxiclav by her GP, after which she noticed a yellowish discolouration of her urine and developed acute epigastric pain, nausea and vomiting. On examination she had icteric sclera, a soft but tender epigastrium and right upper quadrant and was pyrexial at 38.2°C.

## Investigations

### Blood tests

These revealed an obstructive pattern of liver function tests (LFTS):

Gamma-glutamyl transferase: 438 IU l^−1^ (reference range 7–32 IU l^−1^)

Alkaline phosphatase: 398 IU l^−1^ (reference range 30–130 IU l^−1^)

Bilirubin: 61 µmol/L (reference range 0–21 µmol/L)

Alanine aminotransferase: 163 IU l^−1^ (reference range 5–31 IU l^−1^)

She also had a mildly raised amylase (102 IU l^−1^, reference range 0–100 IU l^−1^) and C reactive protein: 27 mg l^−1^ (reference range: 0–5 mg l^−1^) and a mildly reduced lymphocyte count (1.3 × 10^9/L, reference range: 1.5–4 × 10^9/L). An autoimmune liver screen was negative apart from a positive smooth muscle antibody titre of 1/80.

### Infection screens

Urine screen for atypical pneumonia, urinary tract infection, blood cultures, blood-borne virus screen and throat swabs for influenza were all negative.

### Radiology

**Figure 1.**
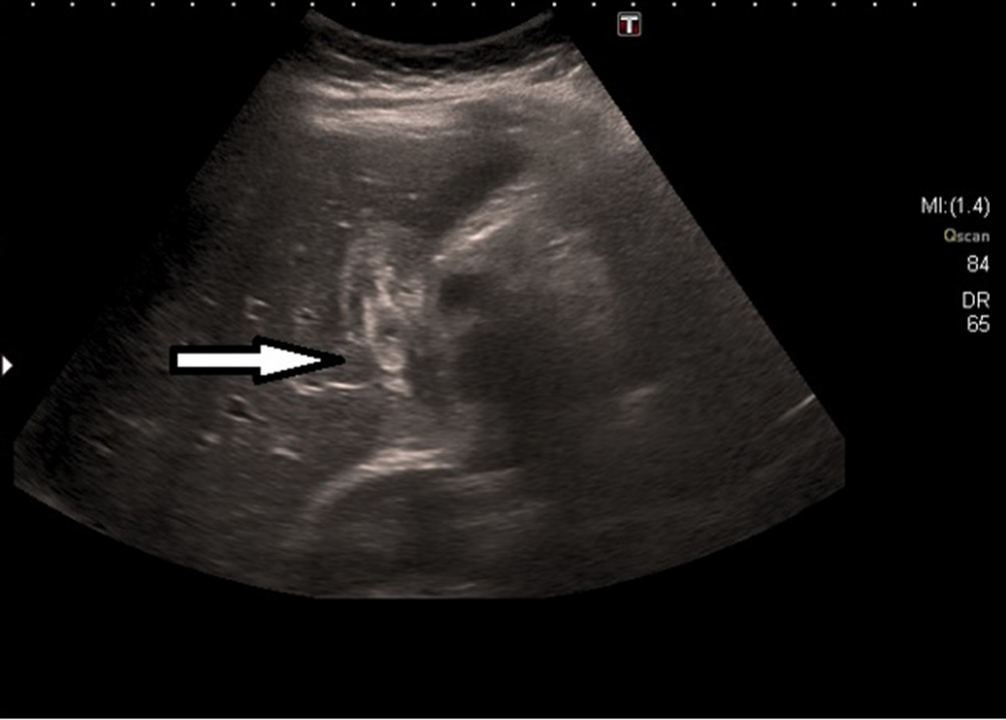
USS abdomen. On retrospective review, changes in keeping with biliary ascariasis were seen. Arrow denotes worm within an oedematous gallbladder with an almost "Bulls’ Eye" appearance. USS,ultrasound scan.

**Figure 2.**
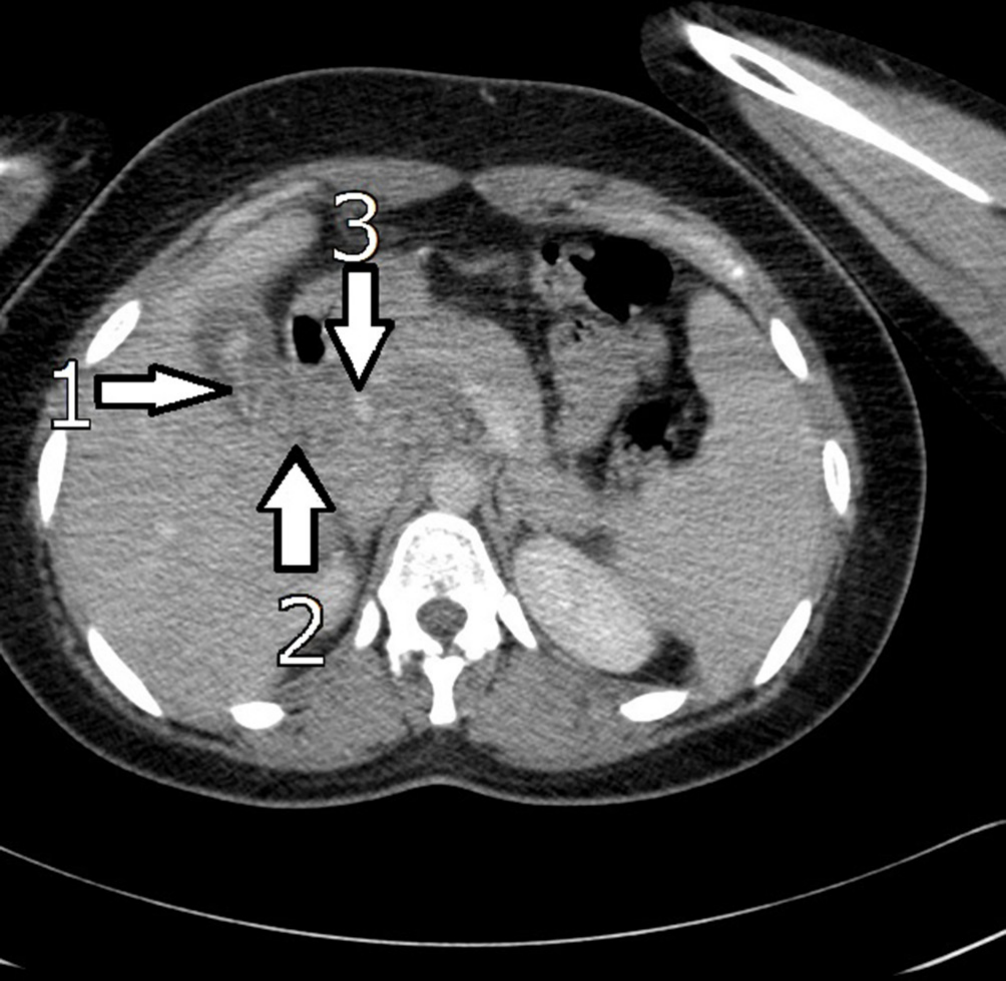
CT abdomen and pelvis with contrast. 1 – high-density signal from worm inside the GB. 2 – worm extending into the proximal CBD. 3 – oedematous pancreatic head. CBD,common bile duct.

**Figure 3.**
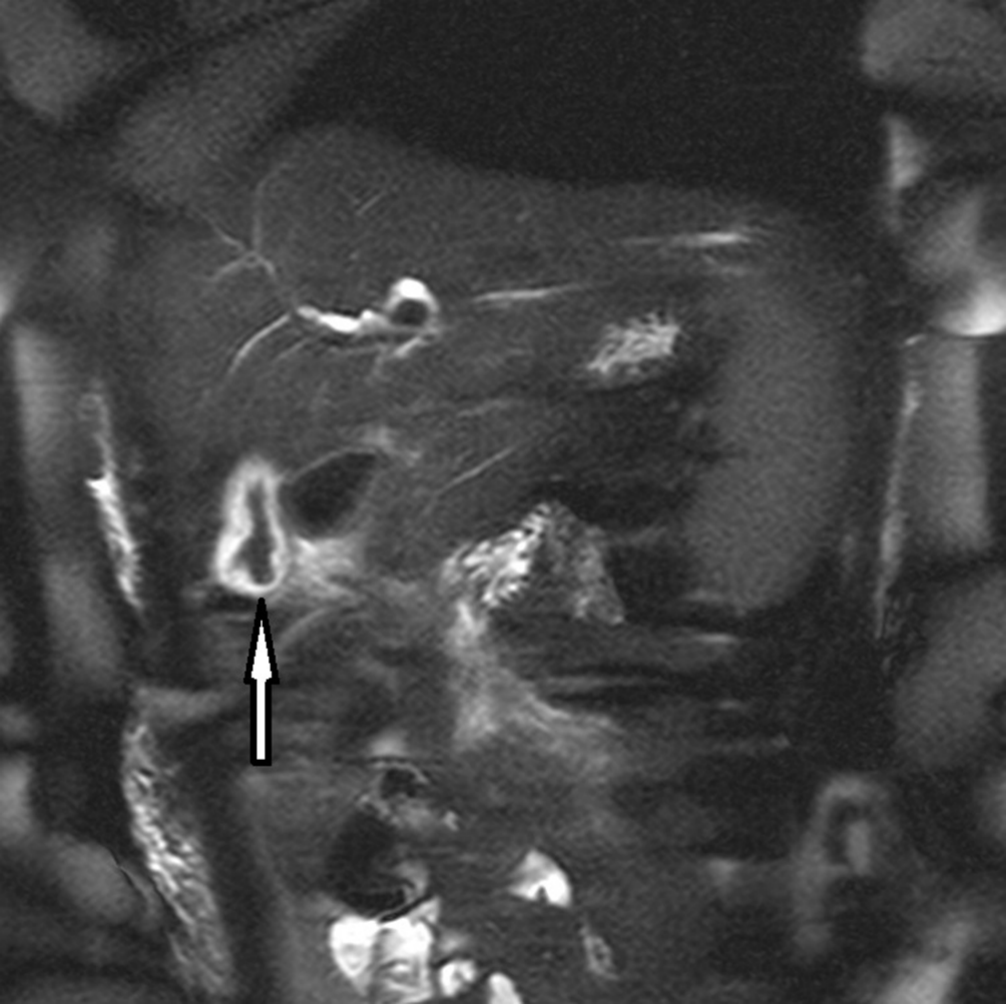
MRCP. Arrow denotes a low-signal roundworm within an oedematous gallbladder. MRCP, MR cholangiopancreatography.

Her chest X-ray (CXR) was clear. An ultrasound scan (USS) of her abdomen showed a thickened, oedematous gallbladder (GB) with hypoechoic areas in the uncinate process suspicious of a degree of pancreatitis (Figure 1). There was however, no convincing evidence of gallstones and a mildly-dilated common bile duct (CBD) at 6 mm. CT of her abdomen and pelvis with contrast showed an oedematous gallbladder with a hyperdense curvilinear structure packed in the GB extending into the CBD, with an oedematous head of pancreas and uncinate process (Figure 2). She went on to have a MR retrograde cholangiopancreatography (MRCP) which confirmed the curvilinear filling defect in the GB and proximal CBD with swelling of the head of the pancreas (Figure 3). On retrospective review of the USS, a worm within the oedematous gallbladder was seen with an almost "Bull’s Eye" appearance, typical of biliary ascariasis^1^ (Figure 1) The most likely differential diagnosis of biliary ascariasis was proposed.

## Differential diagnosis

The patient was initially thought to have: (1) biliary colic due to cholelithiasis and/or co-amoxiclav-induced cholestasis and (2) a viral infection causing her sore throat and fever. However, as her viral throat swabs were all negative and her USS abdomen suggested cholecystitis with a degree of pancreatitis, she was started on intravenous (i.v.) metronidazole, tazocin and fluids for this and admitted under general surgery.

Despite treatment, the patient continued to spike temperatures and had worsening LFTs. With no convincing evidence of gallstones or a source of infection on the initial septic screen, the gastroenterology and microbiology teams were involved. She was given stat doses of gentamicin with every temperature spike. Urine screens for atypical pneumonia and repeat septic screen were all negative. A liver screen was conducted for blood-borne viruses (Human Immunodeficiency Virus, Hepatitis B and C) or an autoimmune cause for her deranged LFTs (primary biliary cirrhosis, primary sclerosing cholangitis and autoimmune hepatitis).^2^ This revealed a positive smooth muscle antibody titre (1/80) which, after discussion with the immunology team, was suggestive of an underlying infection at this titre rather than an autoimmune cause for her worsening LFTs.

The patient went on to have a CT abdomen and pelvis with contrast and MRCP which revealed a thickened gallbladder in keeping with cholecystitis and unusual appearances of the GB and CBD. Upon rediscussion with the radiology team and retrospective review of the USS, the diagnosis of a worm in the biliary tree, most likely biliary ascariasis, given the patient was of Asian origin, with associated pancreatitis was made.

## Treatment

The patient was started on a 3 day course of mebendazole and discharged with follow-up LFTs, USS abdomen and review in outpatient clinic.

## Outcome and follow-up

**Figure 4.**
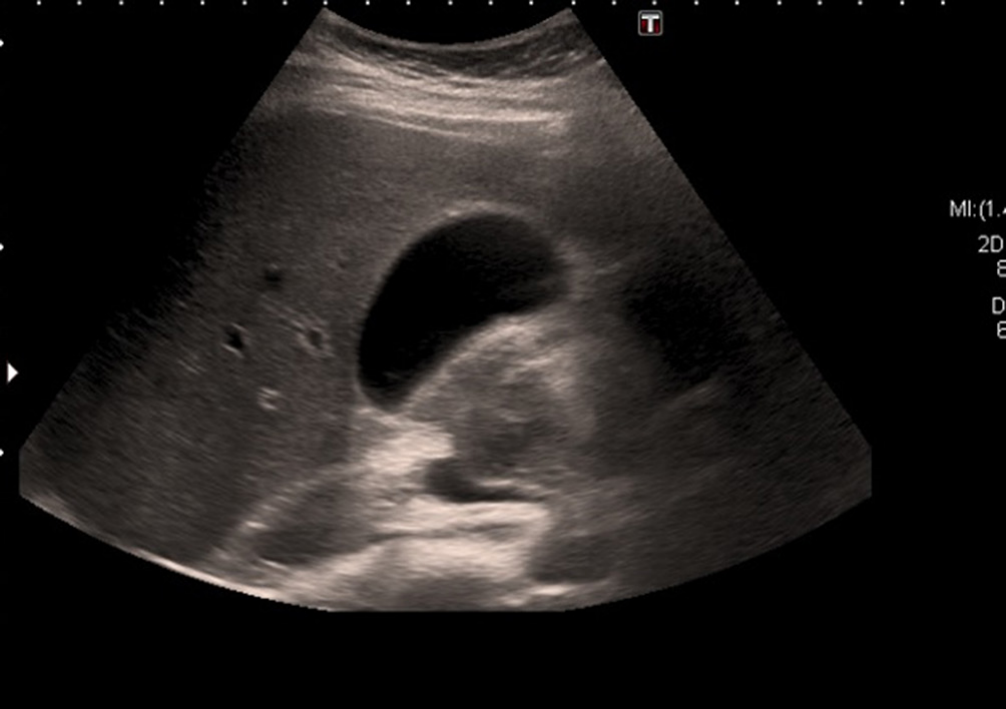
USS abdomen. The gallbladder looks entirely normal. There are no internal filling defects. Normal duct calibre. USS, ultrasound scan.

After 4 weeks, the patients’ LFTs and ultrasound scan (Figure 4) had returned to normal. Her symptoms had completely resolved with conservative management, and she was clinically well. She was thus discharged from clinic and did not require further follow-up or treatment.

## Discussion

### Epidemiology, life cycle and pathology

*Ascaris lumbricoides* is a geohelminth which infects the small intestine (ascariasis) and is one of the most commonly found parasitic infections in the world, affecting over 800 million people, more so in developing countries with poor hygiene and sanitation.^3,4^ Its’ peak prevalence is in children aged 3– to 8 years.^5^

Ascariasis is usually spread by ingesting eggs in infected soil (geophagia) and takes larvae approximately 2 weeks from ingestion of the egg to reach the small intestine via the liver, respiratory tract and oesophagus. Adult male worms can grow up to 15–30 cm in length and females 20–35 cm in length^6^ and live for 1–2 years.^3^

Ascariasis is usually asymptomatic however in large numbers can cause bowel obstruction or migrate to other parts of the body such as the hepatobiliary system giving presentations of biliary colic, obstructive jaundice, pancreatitis and liver abscesses.^6^

### Investigations

Stool microscopy may reveal eggs however may give a false negative result if all male worms present. If ascaris pneumonitis is suspected (caused by the migration of larvae through the respiratory tract), sputum may show larvae and/or eosinophilia. A chest X-ray may show infiltrates.^5^

Imaging such as USS is becoming increasingly common in the diagnosis of biliary ascariasis.^4^ For biliary ascariasis, the recommended imaging is USS, MRCP or endoscopic retrograde cholangiopancreatography (ERCP).^1,7^ A tube-like, rounded or linear filling defect may be seen on ERCP^1,8^ or an echogenic filling defect on USS giving a "Bull’s Eye" appearance. Worm motility may also be seen.^1^ USS can miss up to 50% of infections in the ampulla or duodenum, in which case ERCP is useful.^9^

On CT, a high-density signal may be seen where worms are present, or evidence of pancreatitis suggested by peripancreatic inflammation.^1^

On MRCP, bile would be represented by a high-intensity signal. *Ascaria lumbricoides* would show a low-intensity signal within this with duct dilatation.^1^

The radiological findings can often be misinterpreted for more common pathology such as gallstones (echogenic foci on USS), cholangiocarcinoma (polypoid mass in ducts), bacterial cholangitis (pus which gives a low-intensity signal in the ducts) or recurrent pyogenic cholangitis (intraductal stones leading to duct dilation).^1^

### Treatment

Albendazole and mebendazole are the recommended treatment for ascariasis.^10^ Levamisole and pyrantel panoate can also be used if a water-soluble alternative is needed, *e.g*. the patient is fed via nasogastric tube. Cases which do not respond to conservative treatment can be treated surgically or endoscopically if required.^4,10^

## Learning points

1. Biliary ascariasis should be considered as a cause for cholecystitis and pancreatitis if the patient is not responding to conventional treatment, especially if they are from an endemic area.
2. The recommended imaging for biliary ascariasis is USS, MRCP or ERCP.
3. If the clinical picture is not fitting with the investigation findings you have, or responding appropriately to treatment started, question the initial diagnosis. If in doubt, ask for help!
4. Rarer or more complex presentations of common conditions often require a multidisciplinary approach to achieve the best management plan and outcome for the patient.
